# Independent Learning Module Shows Effectiveness in Improving Preclinical Medical Student Knowledge, Comfort, and Attitudes in Screening and Diagnosis of Eating Disorders

**DOI:** 10.1007/s40596-025-02297-2

**Published:** 2025-12-19

**Authors:** Kira S. Furie, Siena S. Vendlinski, Erin C. Accurso, Zuivanna Rivas, Sophia Collis, Kewchang Lee, Descartes Li, Amanda E. Downey

**Affiliations:** 1 McGaw Medical Center of Northwestern University, Chicago, IL USA; 2 University of South Florida, Tampa, FL USA; 3 University of California San Francisco, San Francisco, CA USA; 4 University of California Davis, Sacramento, CA USA

**Keywords:** Curriculum, Eating disorder, Medical student, Preclinical

## Abstract

**Objective:**

The aim of this study was to develop and assess the effectiveness of an online independent learning module for educating preclinical medical students about eating disorders.

**Methods:**

The curriculum was designed using Kern’s six-step framework. The 60-min eating disorders module incorporated case-based learning, narrative medicine, and real-time feedback to enhance engagement and critical thinking. Surveys were used to assess medical student attitudes, knowledge, and comfort with eating disorder diagnosis and treatment at pre-training and post-training. Paired t-tests and descriptive statistics were used for analysis.

**Results:**

Among 130 preclinical medical students, significant improvements were observed from pre to post-training in diagnostic knowledge (*p* < 0.05). Students also reported significantly improved comfort with eating disorder screening and differential diagnosis (*p* < 0.001), as well as significantly improved attitudes about the importance of medical and psychological support for people with eating disorders (*p* = .008).

**Conclusions:**

Findings provide preliminary evidence of the effectiveness of a brief self-paced independent learning module in improving medical students’ knowledge, comfort, and attitudes in the initial identification and management of eating disorders. Future research can build on these initial findings to examine longer-term knowledge sustainment and additional interventions that may influence the application of knowledge or skills in real-world practice.

Eating disorders (EDs) are life-threatening psychiatric disorders characterized by disturbances in eating behaviors that affect an estimated 5.5–17.9% of women and 0.6–2.4% of men in western settings by early adulthood. [1] Without early detection and effective treatment, EDs can lead to severe outcomes, including malnutrition, organ damage, and death. [2] Furthermore, anorexia nervosa (AN) is one of the deadliest mental health disorders owing to severe medical complications and high suicide rates. [3]

Despite high prevalence and mortality rates, healthcare professionals receive limited education on the recognition and treatment of EDs, leading to delays in diagnosis and treatment. [4, 5] Across countries, medical curricula neglect ED education across all stages of medical training. A survey of all medical schools in the United Kingdom found less than two hours are devoted to EDs over 10–16 years of medical education. [6] A similar survey of residency programs in Canada found that the vast majority of medical residents completed fewer than 5 h of training in ED assessment and treatment since entering medical school. [7] Furthermore, a survey of emergency medicine residents in the U.S. found 93% of residents were unfamiliar with treatment guidelines for patients with EDs, and 85% desired additional training on the care of patients with EDs. [8]

Short ED education modules have been designed to fill the gap in ED training for physicians and students. Multiple types of educational interventions have been shown to improve knowledge, increase comfort, reduce stigma, and enhance attitudes of students and physicians towards individuals with EDs. [9, 10] Similarly, comfort with ED treatment has been found to increase with each additional hour of ED training during residency. [11]

ED-focused educational interventions of various forms have demonstrated effectiveness across multiple stages of medical education. Here we aim to contribute to the growing body of evidence supporting the effectiveness of short, feasible ED-related educational interventions for medical trainees by 1) describing the development and implementation of a brief, self-directed learning module for preclinical medical students, and 2) evaluating its impact on learners’ knowledge, attitudes, and comfort levels in detecting EDs, including AN, atypical AN (AAN), bulimia nervosa (BN), and binge-eating disorder (BED). We hypothesized that completion of the module would yield increased knowledge about EDs and improved comfort with screening and diagnosis.

## Methods

This study was performed at the University of California, San Francisco School of Medicine. Prior to the following intervention, the curriculum included 30 min of formal ED education, focused primarily on medical complications of EDs. Institutional Review Board approval was obtained for this study. The authors followed a six-step approach for curriculum development, as outlined by Kern and colleagues [12], which is described in detail below.

The authors identified substantial evidence highlighting the paucity of ED-related medical education via literature review. The review also revealed various training approaches (e.g., self-paced online module, prerecorded video) that have been effective in improving medical trainees’ and providers’ knowledge about and attitudes toward EDs. Close consultation with medical school leadership identified a significant gap in ED education within the second-year neurology and psychiatry course, which was a feasible setting for the implementation of a new educational initiative. Learning objectives were developed in collaboration with course leadership and aligned with commonly tested criteria on standardized examinations including National Board of Medical Examiners Shelf exams,where ED cases frequently assess diagnosis, medical complications, and management priorities. These objectives were to 1) list diagnostic criteria for AN, AAN, BN, and BED; 2) understand the key aspects of eliciting an ED history applicable to any medical practice; and 3) identify treatment goals for patients with EDs. Further literature review and consultation with course leadership identified an online independent learning module as the most feasible intervention. Online education has been shown to be as effective as traditional, in-person medical student learning models, with additional benefits including enhanced concentration, fewer distractions, greater enjoyment, and improved accessibility. [13] The module incorporated text-based and video resources, a didactic PowerPoint, and case-based learning. Case-based learning has been shown to increase knowledge acquisition compared to traditional lecture methods and is consistently preferred by both faculty and learners. [14, 15] The didactic portion of the module contained information on the fifth edition of the Diagnostic and Statistical Manual of Mental Disorders (DSM-5) [16] criteria for AN, AAN, BN, and BED. Case vignettes were presented following the didactic presentation to help consolidate and synthesize learning. The curriculum was designed to challenge common misconceptions and biases about individuals with EDs through transformative learning by incorporating vignettes with patients across the body mass index (BMI) and gender spectrum. [12, 17] The module addressed both adolescent and adult populations, incorporating broadly applicable content as well as adolescent-specific considerations such as growth curve deviations and family-based treatment, with case vignettes representing both age groups. Students were asked to identify history-taking questions, establish diagnoses, and determine initial medical management. As part of the training, participants were presented with two case vignettes and associated multiple-choice questions. Feedback explaining the rationale for correct and incorrect responses was provided to participants immediately after they answered each question, incorporating metacognition learning principles to enhance critical thinking and self-reflection. [12] Narrative medicine, which has been shown to improve medical student knowledge, clinical skills, and empathy, was integrated throughout videos featuring individuals in ED recovery sharing their lived experiences. [18]

The curriculum was administered entirely via Qualtrics (Qualtrics, Provo, UT) and was designed to be completed within 60 min. All second-year medical students were required to complete the module as a part of their training and had the option to provide informed consent for use of their survey responses for research purposes. To assess the impact of the curriculum, students completed surveys prior to and following the training module, which examined change in knowledge, attitudes, and comfort managing EDs. The Physicians’ Attitude Test was previously used at the study site’s academic medical center to assess knowledge (e.g., *A patient with a clinically significant eating disorder can have a normal BMI*), attitudes (e.g., *Patients with this illness are largely responsible for their own condition*), and comfort with ED diagnosis and treatment (e.g., *How do you rate your comfort in screening patients for eating disorders in your history gathering?*). [5] The survey was administered at pre-training and post-training to examine change in these outcomes. The post-survey included additional questions, including two items from the Workshop Evaluation Form (WEVAL) to assess the usefulness of the course content and students’ satisfaction with the amount of information provided. [19] There was also an open-response section for optional qualitative feedback to gather insights on areas for improvement.

Statistical analyses were conducted using IBM SPSS Statistics (version 29). Analyses focused on evaluating change from pre-training to post-training across individual items assessing knowledge, attitudes, and comfort with ED screening and diagnosis. We used the McNemar test for dichotomous data. For samples in which the number of discordant pairs was fewer than 25, we used the exact binomial version of the McNemar test. For ordinal data (e.g., attitudes), we used the Wilcoxon Signed-Rank Test. Three students mistakenly reviewed the educational module prior to completing the pre-training survey. However, pre- and post-training comparisons did not change in direction or significance when excluding data from these three participants. Therefore, the results reflect analyses that included the full sample.

## Results

Medical students (*N* = 169) completed the educational module in under 60 min, most of whom (*n* = 130, 76.9%) consented to the use of their data for research purposes. Almost all participants (98.5%, *n* = 128) endorsed the content as being useful to them in their capacity as physicians (strongly agree: 71.5%, *n* = 93; agree: 26.9%, *n* = 35) and felt satisfied with having received the right amount of information (95.4%, *n* = 124).

There were significant improvements from pre-training to post-training in several areas of ED knowledge, as well as attitudes towards people with EDs (Table 1). Knowledge decreased slightly with respect to criteria for BED (98.5% accuracy pre-training vs. 93.1% post-training, *p* = 0.039). Participants also reported significantly increased comfort with ED screening and diagnosis from pre-training to post-training (*p* < 0.001).

**Table 1:** Change from pre- to post-training on knowledge and attitudes about eating disorders

	Pre-training	Post-training		
	*n* (%), or *M* (*SD*)	*n* (%), or *M* (*SD*)	*χ*^2^ or *Z*	*p*
Distinguishing between AN^•^ and BN^⋄^	72 (55.4%)	96 (73.8%)	11.500	< 0.001
Knowledge about EDs^γ^ within normal BMI^κ^ range	128 (98.5%)	128 (98.5%)	–-	1.00
BED criteria^λ^	128 (98.5%)	121 (93.1%)	–-	0.039
Comfort with differential ED diagnosis*	0.21 (0.84)	0.91 (0.64)	6.969	< 0.001
Comfort with ED screening*	− 0.05 (0.83)	0.85 (0.66)	8.096	< 0.001
Negative perceptions of people with EDs*	− 1.35 (0.74)	− 1.58 (0.73)	1.633	0.10
Beliefs about urgency and need for treatment*	− 1.21 (0.64)	− 1.43 (0.66)	1.500	0.13
Importance of medical and psychological support*	1.49 (0.64)	1.75 (0.45)	2.646	0.008

^*^Lower scores reflect lower comfort, more negative perceptions, and less strong beliefs about urgency or the importance of treatment

^•^Anorexia nervosa

^⋄^Bulimia nervosa

^γ^Eating disorder

^κ^Body mass index

^λ^Binge eating disorder

The case vignettes highlighted foundational knowledge about some aspects of medical management for EDs and provided important opportunities for corrective feedback and continued learning (Table 2). In response to a case vignette describing a patient presenting with restrictive eating, weight loss, and self-induced vomiting, the vast majority of participants were able to correctly identify appropriate follow-up questions, medical management, and treatment planning steps, but fewer than half correctly identified AAN and AN, binge-purge type, as the two main diagnoses about which to be concerned. However, after receiving corrective feedback about the differences between diagnoses as part of the interactive case vignette, most participants (*n* = 102, 78.5%) were able to appropriately identify that weight was the key factor distinguishing between AN and BN in the context of binge eating and/or purging behaviors. One-fifth (*n* = 26) of participants provided open-text qualitative feedback. The most common comments were expressions of gratitude (*n* = 12), appreciating the integration of didactic slides with the learning module (*n* = 4), and concise, clearly presented content (*n* = 3). Several participants requested an overview handout to reference later to solidify diagnostic differences (*n* = 3). Three participants commented on other aspects of the module they appreciated, including the incorporation of lived experience interviews, the value of reflecting on one’s personal implicit biases and how these may impact patient care, and the case vignettes as being helpful for self-assessment of comprehension.

**Table 2:** Participant responses to a case vignette delivered as part of the training module

Case vignette involving restrictive eating, weight loss, and self-induced vomiting	*n* (%)
*Diagnostic specificity*	
Correctly identified AN-BP^χ^ and atypical AN^•^	61 (46.9%)
Incorrectly identified BN^⋄^	46 (35.4%)
Incorrectly identified AN-R^√^	46 (35.4%)
Incorrectly identified BN	46 (35.4%)
*Appropriate follow-up questions*	
Dietary intake	125 (96.2%)
Weight loss intent	115 (88.5%)
Recent changes in one’s life	116 (89.2%)
*Appropriate recommendations to avoid*	
Do not encourage additional weight loss	126 (96.9%)
Do not encourage additional exercise	121 (93.1%)
*Appropriate management*	
Conduct appropriate medical work-up	123 (94.6%)
Provide diagnostic feedback and psychoeducation	120 (92.3%)
Connection to appropriate medical, mental health, and nutrition support	122 (93.8%)
Case vignette involving binge eating with weight gain	**%** **(*n*)**
*Diagnostic specificity*	
Correctly identified BED^λ^	125 (96.2%)
Incorrectly identified BN	1.5% (2)
Incorrectly identified AN-BP	0.8% (1)
Incorrectly identified OSFED^υ^	1.5% (2)
*Appropriate follow-up questions*	
Overeating episodes	126 (96.9%)
Duration of symptoms	125 (96.2%)
Frequency of episodes	123 (94.6%)
Intent to lose weight or prevent weight gain	106 (81.5%)
*Appropriate recommendations to avoid*	
Do not refer to weight loss program	129 (99.2%)
Do not refer to overeaters anonymous	129 (99.2%)
Do not use language that might increase shame (e.g., “Have you tried buying healthy snacks instead of junk food?”)	125 (96.2%)
Do not encourage additional exercise	130 (100%)
*Appropriate management*	
Provide diagnostic feedback, psychoeducation, and connection to appropriate medical, mental health, and nutrition support	98.5% (128)

^χ^Anorexia nervosa, binge-purge subtype

^•^Anorexia nervosa

^⋄^Bulimia nervosa

^√^Anorexia nervosa, restricting subtype

^λ^Binge eating disorder

^υ^Other specified feeding or eating disorder

## Discussion

Our results suggest a 60-min, case-based, online learning module for preclinical medical students had good perceived appropriateness and satisfaction. Participants who provided qualitative feedback largely expressed appreciation for the content. Further, the module was effective in increasing knowledge and comfort in addressing EDs with the exception of a question on BED. This may have occurred due to increased knowledge on the definition of a binge eating episode without adequate attention paid to the criterion of patients’ experiences of a sense of distress or loss of control.

While medical students’ comfort with ED screening and diagnosis greatly improved when comparing pre- to post-survey data, the analysis of vignette responses revealed ongoing challenges in accurate diagnosis. This raises a broader question about the primary aim of educational interventions for preclinical medical students. While U.S. medical schools are obligated to prepare students for national exams that test ED knowledge through the lens of DSM-5 diagnostic criteria, accurate diagnosis of specific ED subtypes is rarely the most salient clinical knowledge for healthcare providers. Given high diagnostic crossover and overlapping treatment approaches, a meaningful educational objective may be the ability to broadly detect an ED, recognize medical and psychological sequelae, and support individuals in obtaining continued specialized care. [3]

The lack of standardized medical education provided about EDs likely contributes to the discomfort many physicians feel when addressing EDs. A 2020 study of residents and fellows across various medical disciplines found that only 11.4% reported feeling comfortable managing patients with EDs, though comfort significantly improved with experience treating such patients. [5] While an online module cannot substitute for direct patient experience, our findings support a growing body of literature that suggests introducing brief ED-focused educational material early in training can enhance medical trainee comfort and confidence in future clinical scenarios when identifying and caring for an individual with an ED. [20]

This study has several limitations. First, this study did not use a randomized design, and it did not evaluate the longer-term knowledge retention or the impact of improvements in knowledge and attitudes on real-world clinical practice. Further, the case vignettes did not include pre-training questions, limiting our ability to assess the effectiveness of each case in improving case-specific clinical reasoning. Future iterations of this curriculum will address these limitations by using a randomized or other experimental design with refined pre- and post-survey questions. Further, although our cases reflected a range of DSM-5 diagnoses, future module development will strengthen practical competencies including recognition of core ED warning signs and the ability to triage appropriately as well as their impact on knowledge retention and clinical application across longer-term follow-up periods.

This study also has several notable strengths. Regarding study design, the pre- and post-test methodology allowed for prospective analysis of changes in knowledge and comfort levels following exposure to the module. Integration into required coursework resulted in a high rate of participant engagement, with over 75% of the class consenting to the use of their data. Regarding the module itself, the online, self-paced format facilitated engagement with the material at students’ convenience without requiring additional faculty time or physical space. The incorporation of immediate feedback promoted engagement and knowledge retention in real time. [12, 13] Patient-centered perspectives on ED care further enriched the learning experience.

We found evidence to support the appropriateness, satisfaction, and effectiveness of a 60-min independent learning module in improving preclinical medical students’ knowledge, attitudes, and comfort with screening for and diagnosing EDs. A short, independent learning module may be a feasible, inexpensive, and transportable method to introduce critical ED-related content into medical school curricula. Future educational modules may emphasize that an initial focus on establishing regular, structured eating patterns is a unifying principle across evidence-based treatments for eating disorders. Future studies may evaluate the applicability of the revised module at other medical schools and evaluate its long-term effectiveness.

## Data Availability

The data that support the findings of this study are not openly available due to reasons of sensitivity and are available from the corresponding author upon reasonable request. Data are located in controlled access data storage at the University of California, San Francisco.
